# Revisiting the Role of Public Opinion in Foreign Policy: The Case of Brunei in the South China Sea

**DOI:** 10.12688/f1000research.177157.1

**Published:** 2026-03-19

**Authors:** Bama Andika Putra

**Affiliations:** 1 University of Bristol School of Sociology Politics and International Studies, Bristol, England, UK; 2 Universitas Hasanuddin Fakultas Ilmu Sosial dan Ilmu Politik, Makassar, South Sulawesi, Indonesia

**Keywords:** South China Sea, Brunei Darussalam, Public Opinion, Southeast Asia, Foreign Policy

## Abstract

Although claiming a rectangular Exclusive Economic Zone (EEZ) that encompasses the Louisa Reef and maritime features in the Spratly Islands, Brunei has not taken a stance that reflects decisiveness in safeguarding its claims in the South China Sea. For decades, scholars have argued that economic factors fuel Brunei’s silence in the disputed waters. However, as a means of seeking an alternative interpretation, this study argues for the relevance of the nexus between public opinion and foreign policy and perceives that Brunei’s stance can also be understood through the lens of how the Brunei people perceive the South China Sea dispute. Utilizing published data from the State of Southeast Asia 2025 survey report (with slight comparisons with the 2024 survey), the qualitative analysis concludes the following: 1) Socio-demographic factors and conceptual schemes/belief systems as influencing variables in shaping the Brunei public’s foreign policy attitudes, by acknowledging the multi-dimensional factors associated to the South China Sea dispute; and 2) the top-down model to explain the interaction between public opinion and foreign policies, with Brunei respondents adopting a similar stance to the Sultanate through the cautiousness express over the disputed waters, expressed ongoing trust towards regional mechanisms, as well as the favorable perception towards China from the lens of influential economic powerhouse, and strategic significance in Southeast Asia.

## 1. Introduction

**Figure 1.**
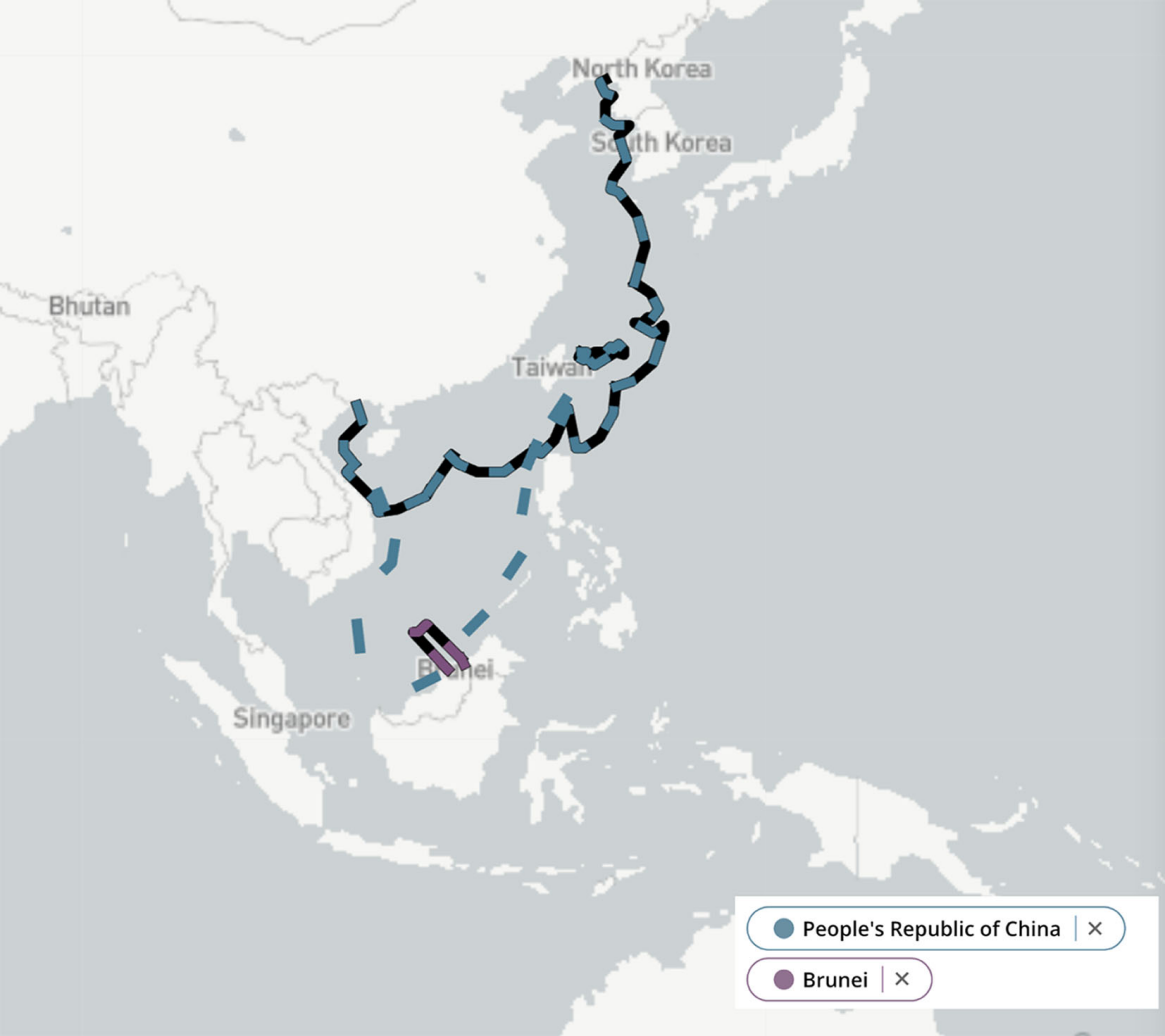
Comparison of China and Brunei’s Exclusive Economic Zones. Source: Adapted from the Asia Maritime Transparency Initiative ( AMTI, 2023a).

In 2025, Brunei Darussalam and China decided to deepen their cooperation in oil and gas exploration in the South China Sea. As multiple sources reported, their cooperation marks the continuation of the joint venture set up between the Brunei National Petroleum Company and the China National Offshore Oil Corporation, as well as the adoption of the terminology of ‘mutually agreed areas’ during Sultan Hassanal Bolkiah’s visit to Beijing ( Bimo, 2025; Yilmaz, 2025; Zhou, 2025). To provide context, this joint venture is significant within the discourse of the South China Sea dispute. Brunei is a claimant state towards the disputed waters, claiming parts of the Louisa Reef and several maritime features in the Spratly Islands ( Espena & Uy, 2020; Hart, 2018; Jacques, 2018; Sands, 2026). Brunei’s rectangle-shaped Exclusive Economic Zone (EEZ) (see Figure 1), however, has not been safeguarded with decisiveness by the Sultanate.

In the past decade, there have been connections made between Brunei’s intentions of diversifying the state’s economy and the stance it takes in the South China Sea dispute ( Druce & Julay, 2019; Elleman, 2017; Hart, 2018; Jacques, 2018; Putra, 2021; Slesman & Baha, 2023; Tisdell, 1998). As a means to reduce dependence on raw resource exports, Brunei has sought assistance from China and linked Brunei’s *Wawasan Brunei 2035* (Brunei's long-term grand strategy) to China’s Belt and Road Initiative, with the hope that convergence in grand strategies will lead to a more economically sustainable Brunei ( Bo, 2017). Therefore, for Brunei, the issue of the South China Sea involving China is not independent of other influential factors shaping Brunei’s South China Sea policy.

**Figure 2.**
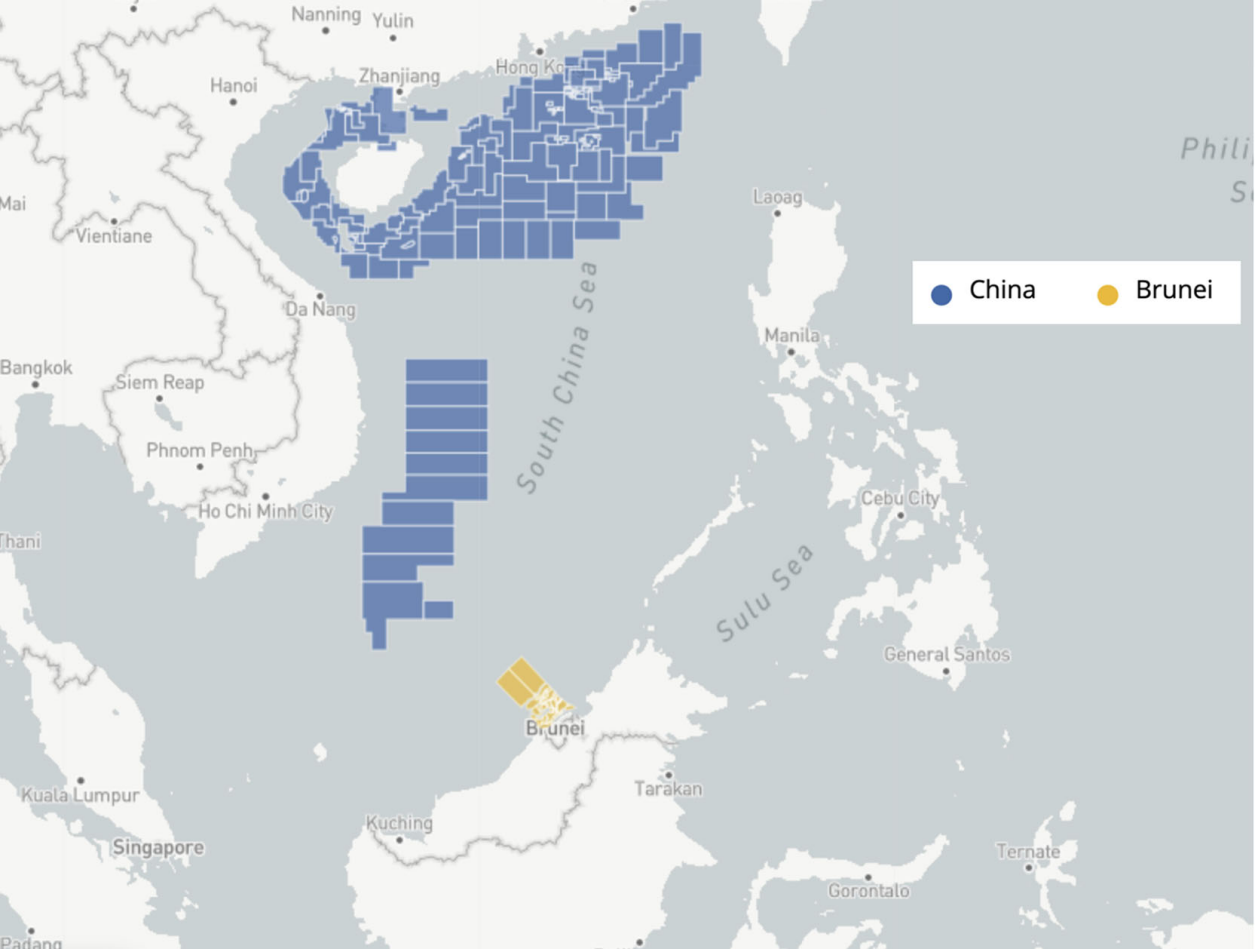
Claimed oil and gas blocks in the South China Sea between Brunei and China. Source: Adapted from the Asia Maritime Transparency Initiative ( AMTI, 2023b).

How has the existing literature understood Brunei’s stance in the South China Sea? As reported by the Asia Maritime Transparency Initiative, there have been increasing instances of Chinese law enforcement vessels intruding into Brunei’s EEZ ( AMTI, 2020). What is interesting, however, is how Brunei responds to these incursions. Unlike the other claimant states to the South China Sea that displays a heavy presence of coast guards, fishing militias, and fisheries surveillance vessels within their claimed EEZ in the South China Sea ( Chubb, 2022; Ha, 2019; Hong Hiep, 2019; Lagniton, 2025; Sangtam, 2021; Simonette, 2023; Sulaiman, 2025; Zattullah et al., 2021), uniquely, Brunei has not taken a similar pathway. Brunei does not adopt a policy reflecting a heavy presence of its maritime constabulary forces within its claimed EEZ, which strikes as confusing compared to those of the other claimant states to the disputed waters. As shown in Figure 2 below, Brunei’s oil and gas exploration in the South China Sea is the only waterway it can exploit to boost its oil- and gas-dependent revenues. However, why does the Sultanate display a greater leaning towards collaborative efforts, rather than confrontational ones?

The majority of studies in the past have claimed that Brunei Darussalam’s South China Sea policy is represented by ‘silence’ ( Espena & Uy, 2020; Jacques, 2018; Kurniati et al., 2025; Putra, 2021, 2024; Sands, 2026). These studies have concluded that Brunei’s interests in the South China Sea exceed the considerations of sovereignty within its maritime borders. Instead, there is a thick economic discourse attached to its decision, leading Brunei to adopt a stance different from that of the other claimant states. However, this has been the primary interpretation for more than a decade. Is there another angle of interpretation that can be made that goes beyond the assertion that economic factors prevail?

To make sense of Brunei’s silence and relatively calm response to China’s challenges in the South China Sea, this article takes a different angle, highlighting the role of public opinion in foreign policy. It takes the task of measuring and delineating the relationship between public opinion and foreign policy decisions, bridging the conceptions introduced by Jean-Christophe Boucher in 2024. Two of Boucher’s ideas are bridged for this study: 1) The relevance of socio-demographic factors and conceptual schemes, as influencing factors in the public’s foreign policy attitudes; and 2) the applicability of the ‘top-down’ interpretation, in which elites shape the public’s preferences and opinions over foreign policy matters ( Boucher, 2024).

In doing so, this qualitative study uses published data from the ISEAS-Yusof Ishak Institute’s State of Southeast Asia Report in 2024 and 2025 ( Seah et al., 2024, 2025) and examines how Bruneians perceive China and the South China Sea. The 2025 survey included 2,023 respondents from the ten Association of Southeast Asian Nations (ASEAN) member states, with citizens of Brunei accounting for 7.5% of the total. To isolate and reveal Brunei’s public opinion, only answers from Brunei citizens will be considered for this study, specifically in the sections on regional outlook on international developments, major power influences, US-China rivalry in Southeast Asia, and perceptions of trust. Meanwhile, the argument over the applicability of public opinions within the context of foreign policies has been argued in past studies, which shows the evolving role of public opinion and the vast dynamics related to it, with foreign policy decisions ( Fearon, 1998; Rosenau, 1961; Snyder et al., 1954; Sprout & Sprout, 1956). Different, however, from past studies that draw upon the nexus between public opinion and foreign policy, this study tests those assumptions in the context of an absolute monarchy like Brunei Darussalam, where a top-down hierarchy has been apparent for decades and is centered on the authority of the Sultanate.

## 2. Brunei in the South China Sea: A literature review

Discussion on Brunei in the South China Sea is multidimensional and not confined to a single discourse. Consequently, this literature review section will explore the three most relevant discourses related to the discussions of this study. They include Brunei’s perceptions of the importance of the South China Sea, the emergence of the terms ‘silent’ and ‘vanishing’ claims in the disputed waters, and the nexus between the disputes and Brunei’s bilateral relations with China. Doing so allows readers to gain a glimpse of the factors that influence Brunei’s decision-making. It makes it possible to argue the potential applicability of public opinion discourses to make sense of Brunei’s puzzling stance in the South China Sea.

At the core, one of the central discourses concerns the significance of Brunei’s South China Sea claims over the Louisa Reef and several maritime features in the Spratly Islands. As Elleman’s 2017 study showed, the hydrocarbon resources that are starting to deplete have led Brunei’s leaders to seek alternative resources that could be exploited, and the maritime zones within its EEZ are among them ( Elleman, 2017). Nevertheless, past studies have also argued differently. Although there is acknowledgement that this resource is significant, there are also arguments stating that Brunei’s South China Sea claims are not as substantial as those perceived by other claimant states ( Espena & Uy, 2020; Hart, 2018; IMOA, 2020; Kurniati et al., 2025; Putra, 2021, 2024; Sands, 2026).

Based on that context, the second discourse on Brunei’s silent claims in the South China Sea becomes essential to evaluate. In several of the author’s past published studies ( Putra, 2021, 2024), the argument is that China has adopted a silent claim in the disputed waters due to increasing dependence on China’s Belt and Road Initiative (BRI) and expectations of expanding trade relations. These build on past arguments that have concluded that Brunei’s stance is unique, due to its policies that tend to run counter to the usual policies of Southeast Asia’s claimant states. Kurniati, Laksono, and Aulia’s study, for example, noted that the convergence of interests between Brunei and China has led Brunei, “[…] challenging ASEAN’s unified action on the South China Sea dispute” ( Kurniati et al., 2025, p. 66). Observing this trend, Sands also argued that this has been the reason Brunei is the only claimant state in the South China Sea without a military presence in the Spratly Islands ( Sands, 2026).

However, some argue that Brunei is adopting other approaches that do not quite fit the term ‘silent claim’. As these studies argue, the point is that Brunei is balancing between different approaches, including engaging with ASEAN to seek solutions and continuing to advocate for an international-law-based resolution to the tensions ( Espena & Uy, 2020; IMOA, 2020; Wei, 2024). In Wei’s 2024 study, for example, he concluded that Brunei’s South China Sea policy is ‘calculated,’ by intentionally creating “[…] specific relationships with these major powers (the US and China); so long as its territorial waters remain undisturbed” ( Wei, 2024).

As discussed, there appear to be other causal factors that determine Brunei’s unique stance in the South China Sea. This is why the third discourse on Brunei’s relations with China, specifically in the economic domain, is influential in making sense of the empirical anomaly. Past studies have interpreted the arrival of the BRI as a golden moment for Brunei, as the nation opens up to measures that allow Brunei to diversify the state’s revenue sources ( CSPS, 2022; Koh, 2024; Loon, 2025; Sands, 2026; Slesman & Baha, 2023; Tisdell, 1998). The key point here is Brunei’s intention to diversify away from the oil and gas sector toward alternative sectors that allow the nation to develop ( Hashim et al., 2025; C. Y. Hoon & Zhao, 2023). It is no surprise that Brunei has been connected with a favorable and receptive stance after the arrival of the BRI. As Lawrence mentioned in 2021, “The convergence between the Sultan’s Brunei Vision 2035 ( *Wawasan Brunei 2035*) and Xi Jinping’s BRI has increased the political importance of Chinese foreign direct investment (FDI) to Brunei. Thus, embracing the BRI is key to the elite’s development-based performance legitimation” ( Lawrence, 2021). Similarly, Lim, Hoon, and Zhao also argued in alignment with this by stating, “Faced with dwindling oil and gas reserves, Brunei has been hard-pressed to diversify its reliance on hydrocarbon […] China has emerged as an attractive prospect to the Brunei government” ( Lim et al., 2023, p. 242).

Although it seems that, from an alignment perspective, Brunei aligns with China, this is in fact at odds with another body of studies on Brunei’s foreign policy. Perhaps related to all three discourses discussed, a large number of studies have used the term ‘hedging’ to explain Brunei’s foreign policy in contemporary times. Hedging, by definition, is a middle position between balancing and bandwagoning ( Goh, 2016; Haacke, 2019; Jones & Jenne, 2022; Marston, 2023). Within the context of Southeast Asia, Brunei has often been associated with this term to signify the Sultan’s alignment with the interests of both the US and China, simultaneously ( Dayant & Stanhope, 2025; Husseini, 2023; Kuik, 2021; Short, 2025; Tumala, 2025).

Nevertheless, a deficiency in these discourses is the lack of explanatory depth into why this empirical puzzle of Brunei’s unique stance in the South China Sea has surfaced. The conclusions drawn in past studies tend to be generalist about the economic sector, without delving deeply into the variables within the state that also holds great importance to understand Brunei’s South China Sea policy. To close the gap in this literature, the following section will explore the significance of public opinion in the context of foreign policy and introduce the two analytical frameworks that will be utilized.

## 3. The proposed analytical framework: Revealing influencing factors and measuring interactions of public opinions and foreign policy

The significance of public opinion within international relations is well documented in past studies. Realist scholars, for example, have argued that public opinion does affect a state’s foreign policy ( Christensen, 1996; Schweller, 1998; Wolfers, 1952; Zakaria, 1998). Arguing for the significance of agents, studies labelled as constructivist have also pursued a similar research inquiry, examining the role of public opinion in the construction of norms and identities ( Finnemore, 2013; Kahl, 1998; Katzenstein, 1996). Consequently, there may be different angles for interpreting the role of public opinion in foreign policy. For this study, the definition provided by Boucher in 2024 serves as a good starting point: “[…] public opinion represents views, beliefs, attitudes, or preferences found amongst the public […] such preferences can be widespread or specific to certain interest groups, learned or uninformed, mobilized or latent, a settled belief or volatile” ( Boucher, 2024, p. 251). Looking beyond categorizing public opinion studies into one of the international relations camps or the other, studies in the past have differed according to the discourses asked about the public opinion’s role in foreign policy.

Several waves of studies in public opinion and foreign policy can be traced. The first, for example, is a group of studies that have concluded that public opinion is irrational and therefore rarely influences foreign policy ( Carr, 1936; Converse, 1964; Lippmann, 1955). After the Vietnam War and the resurgence of public opinion’s role in shaping foreign policy, a new wave of inquiry emerged. This would later reveal that there are circumstances in which public opinion could be stable ( Holsti, 1996; Mueller, 1973; Page & Shapiro, 1992; Shapiro & Page, 1988). The claim is, therefore, that public opinion is rational, and society has the capacity to make sound judgments on foreign affairs ( Boucher, 2024; Neuman, 1986; Page, 1996; Powlick & Katz, 1998).

Against the backdrop of studies on public opinion in foreign policy, two of Boucher’s introduced analytical frameworks are utilized in the study of Brunei’s public opinion vis-à-vis the South China Sea. The first focuses on factors influencing foreign policy attitude. As Boucher mentions, this focuses on identifying the causal mechanisms that influence public opinion’s attitudes towards foreign policy, which could be based on several variables identified in past studies. These include socio-demographic factors, influence of political ideology, foreign policy beliefs/conceptual schemes, as well as micro-foundations ( Conover & Sapiro, 1993; Domke et al., 1987; Eichenberg, 2019; Holsti, 1962; Holsti & Rosenau, 1996; Inglehart, 2000; Kertzer, 2017; Kleinberg & Fordham, 2018; Kreps & Maxey, 2018; Levi, 1970; Milner & Tingley, 2015; Paxton & Knack, 2012).

Nevertheless, in the context of Brunei’s foreign policies and the data available to the author, only several of Boucher’s specified variables can be utilized. Regarding the socio-demographic factors, the arguments introduced are based on respondents’ age and education. Therefore, although there is a considerable number of studies arguing that gender, for example, influences the view of respondents towards foreign policy ( Conover & Sapiro, 1993; Eichenberg & Stoll, 2017; Kleinberg & Fordham, 2018; Togeby, 1994), the limitation of datasets limits the capacity of this study to argue that point. It is expected, however, that the data publicly available in ISEAS-Yusof Ishak Institute allows this study to claim whether more educated respondents favor more exposure towards trade and internationalism or not, as was argued in past studies ( Domke et al., 1987; Kleinberg & Fordham, 2018; Paxton & Knack, 2012).

The second influencing variable is conceptual schemes/belief systems and micro-foundations. It is argued that, as individuals, perceptions are directed toward making the world fit the perspectives of a particular individual ( Boucher, 2024; Holsti, 1962). In the case of the surveys in 2024 and 2025, the available dataset can be understood as related to this variable through the lens that the institutional backgrounds of the respondents tend to direct and isolate the way the issue of the South China Sea is perceived. Similarly, the variable of micro-foundations can be understood as moral values embraced by individuals ( Bayram, 2017; Goren et al., 2016; Kreps & Maxey, 2018; Rathbun et al., 2016; Schwartz, 1992). Meanwhile, several of Boucher’s identified influencing variables will be excluded: gender, political ideology, and micro-foundations (moral values). The reason for this is that the dataset does not make these variables clear; for example, the last two variables (political ideology and micro-foundations) were not asked in the surveys.

To understand the nexus between public opinion and foreign policy, a second analytical framework is introduced to identify and measure their interaction. Of the three models introduced in Boucher’s 2024 study ( Boucher, 2024), the case of Brunei in the South China Sea is best suited to the first approach, the top-down model. The top-down model argues that public opinion is shaped by political elites, defined as “politicians, higher level of government officials, […] (and) experts and policy specialists” ( Zaller, 1992, p. 6). Foreign policy issues are far beyond the reach of citizens; therefore, this model shows that the public relies on elites’ views as a heuristic shortcut to understanding foreign policy affairs ( Boucher, 2024; Western, 2005; Wittkopf, 1994; Zaller, 1992).

Why is the top-down approach most applicable in the case of Brunei in the South China Sea? Unlike many past studies on the role of public opinion in foreign policy, Brunei is not a democratic country. The absolute monarchy system adopted, therefore, entails a process in which the Sultanate is solely responsible for the major direction of Brunei Darussalam’s foreign policies. In the context of the South China Sea, which is understood as a high-profile case and one involving the interests of the Sultan to realize the *Wawasan Brunei 2035* through the financial incentives offered by China, it is without a doubt that Sultan Hassanal Bolkiah is interested in shaping the perspectives of the Brunei society to control for potential negative perspectives towards China. Looking at the State of Southeast Asia reports in 2024 and 2025, this framework allows this study to conclude that there is convergence in perceptions between those held by the Sultan and those expressed in public opinion through the survey reports.

## 4. The alternative interpretation proposed: Brunei’s public opinions of the South China Sea and its impact on foreign policy

To reveal public opinion among Brunei citizens regarding the South China Sea, this section will be structured as follows. First, it provides information on who the respondents were in the 2025 survey report, including variables such as age and education, to provide background on the respondents. Second, it elaborates on several key findings and opinions under the themes of regional outlook on international developments, major power influences, US-China rivalry in Southeast Asia, and perceptions of trust, which are correlated with the dynamics in the South China Sea. Third, through Boucher’s analytical frameworks that reveal factors influencing foreign policy attitude (age, education, and conceptual schemes/belief systems) and form of interaction between the public’s foreign policy opinions and decision makers, this study explores how these two frameworks are applicable in understanding the position and role of public views in the context of Brunei’s South China Sea policy. Within this framework, this study measures and delineates the relationship between public opinion and the eventual foreign policy direction taken by Brunei.

### 4.1 Revealing opinions on the South China Sea: Factors influencing Brunei’s public opinions in the South China Sea

In the ninth year of the State of Southeast Asia survey, a better understanding of how the public perceives geopolitical developments is made possible by selecting respondents who are in a position to inform or influence policy. The total number of respondents was 2,023 from the ten ASEAN members and Timor-Leste ( Seah et al., 2025). The respondent’s background varied across several affiliation categories: academia, think tankers/researchers, private sector representatives, civil society/non-governmental organizations/media representatives, government officials, and regional/international organization personnel ( Seah et al., 2025, p. 3). In conclusion, Brunei’s respondents accumulated to 7.4% of the total respondents. However, for this study, only responses from Brunei-linked affiliations will be considered representative of the voices of Brunei citizens. The socio-demographic features of the respondents, along with their affiliations, are presented in the following table.

As seen in Table 1, the respondents’ socio-demographic backgrounds are diverse, providing a comprehensive perspective. The majority of respondents have Bachelor’s and Master’s Degrees, indicating that they are well educated and capable of making sound decisions and providing answers. Meanwhile, the majority of respondents’ background affiliations were from the private sector, with a group age distribution equally dominant between 22 and 60 years old. These factors are integral to understanding Brunei’s public opinion on the South China Sea, given the potential for critical responses. It also shows that these variables would most likely influence the conceptual schemes/belief systems adopted by the respondents. This can be seen, for example, in the respondents’ affiliation categories. Those in the private sector, specifically in business and finance, would view the South China Sea primarily through an economic lens, focusing on how developments in disputed waters would affect the nation’s economy. Those with a higher level of education are also expected to consider multiple factors affecting the South China Sea, rather than being limited to single concerns.

**Table 1.** Highest education level, affiliation, and age group of respondents (percentage of all brunei respondents).

Education	Post-Secondary/Non-Tertiary	4.7%
	Bachelor’s Degree	43.6%
	Master’s Degree	39.6%
	Doctoral Degree	12.1%
Affiliation	Academia, Think-Tanks, or Research Institutions	18.8%
	Civil Society, Non-Governmental Organizations, or Media	10.7%
	Government	21.5%
	Private Sector (Business or Finance)	40.3%
	Regional or International Organizations	8.7%
Age Group	18–21	2.0%
	22–35	30.9%
	36–45	32.2%
	46–60	33.6%
	61–69	1/3%

Source: Adapted from the State of Southeast Asia 2025 Survey Report ( Seah et al., 2025).

The complexity of the perspectives and views of the Brunei people is well documented in survey reports that ask for more general opinions on international developments that influence Brunei Darussalam and the Southeast Asian region in general. For example, in the section of ‘regional outlook and views on international developments,’ Brunei respondents did not favor a challenge affecting Southeast Asia that was more dominant compared to others. Several of the answers were selected by more than 50% of the respondents (based on percentage), which includes the challenges of intensifying economic tensions among major powers, unemployment, and increased military tensions (regionally in Asia) ( Seah et al., 2025). Compared to 2024, the perceived challenges are more widespread, as respondents in 2024 selected unemployment and widening socio-economic gaps as the main challenges encountered in Southeast Asia ( Seah et al., 2024).

In a more specific question, respondents are pushed to select three geopolitical events that are the primary concern of their country. Brunei’s public opinion in the 2025 report showed that two geopolitical events have been the main concerns for the Brunei Government: the war between Israel and Palestine (56.4%) and the aggressive behavior in the South China Sea (57.7%) ( Seah et al., 2025). A unique observation is that respondents who selected the South China Sea issue were mainly from the ASEAN claimant states, including the Philippines (90.3%), Vietnam (74.8%), and Brunei (57.7%) ( Seah et al., 2025, p. 16). Nevertheless, these figures from 2025 show that Brunei citizens’ perception of the significance of aggressive behavior in the South China Sea is significantly lower than that of other claimant states.

Meanwhile, one of the figures in the section on ‘major powers’ regional influence and leadership provides some unique insights into Brunei’s perceptions. When respondents were asked which country/organization they have confidence in maintaining a rules-based order, surprisingly, China has increased in the percentages. The increased percentages are significant enough that it is now perceived as the third actor that Brunei citizens have the most confidence in upholding international law, after ASEAN and the US. This is a significant point in understanding the general perception of the Brunei people, considering that Brunei does have an official disputed EEZ with China, and that China has been attached with the discourses of going against the provisions of international laws ( Elleman, 2017; Espena & Uy, 2020; IMOA, 2020; Putra, 2024; Sands, 2026; Xinhua, 2016). Therefore, although a high confidence is expected with ASEAN and the US, China is a surprising answer. A comparison of the percentages from the 2024 and 2025 surveys is shown in Table 2 below.

**Table 2.** Confidence in the actors to provide leadership to maintain the rules-based order (2024 and 2025 comparison in percentages, Brunei respondents).

	The State of Southeast Asia 2024 survey report	The State of Southeast Asia 2025 survey report
ASEAN	40.3%	20.8%
Australia	1.3%	6.0%
China	11.7%	16.8%
The European Union	13.0%	13.4%
India	0.0%	3.4%
Japan	10.4%	11.4%
New Zealand	5.2%	0.7%
Republic of Korea	2.6%	4.0%
The United Kingdom	3.9%	4.7%
The United States	11.7%	18.8%

Source: Adapted from the State of Southeast Asia 2025 and 2024 Survey Report ( Seah et al., 2024, 2025).

Several survey results from 2024 and 2025 reveal unique insights that allow this study to examine the relevance of several variables in shaping the Brunei public’s perceptions. The first noticeable thing is that respondents do not downplay issues related to China and the South China Sea, indicating they acknowledge that a tension exists and poses a challenge for the Brunei Government. Those general perceptions themselves mimic that of the Sultanate, in which Brunei confronts multiple security challenges in the region, and the South China Sea is only one among many ( Kurniati et al., 2025; Lee et al., 2024; Shaw, 2016; Widyawardhana et al., 2018). Furthermore, the respondents also express that the South China Sea issue is present but not a significant concern, unlike the voices expressed in the cases of Vietnam and the Philippines. This is also a similar stance to the Sultan, which perceives that the South China Sea is a challenge, but does not require excessive responses in the form of populating the disputed waters ( IMOA, 2020; Kurniati et al., 2025; Noor & Daniel, 2016; Putra, 2021; Roach, 2014; Xinhua, 2016; Zhou, 2025).

Regarding the variables that influence this, several conclusions can be made. As argued with Boucher’s analytical framework, the socio-demographic factors are influential in the respondent’s perceptions as they are variables that are “[…] proxies for a deep-seated socialization process and gained experience which shapes attitudes on policy issues, and results are relatively consistent across foreign policy issues” ( Boucher, 2024, p. 252). Seen, for example, how Brunei citizens express a relatively equal concern for different geopolitical challenges in Asia, which are not confined to the issue of the South China Sea, the socio-demographic variables of education and affiliation background yield a balanced perception among Brunei citizens.

Given the variable of conceptual schemes/belief systems, as argued previously, there is a likelihood that respondents frame the questions to fit their images and understandings ( Boucher, 2024; Holsti, 1962). The relatively balanced perception of Brunei citizens can be attributed to the fact that, although incursions by Chinese law enforcement vessels occurred in the past ( AMTI, 2020), this is not a continuing development that warrants a sustained threat perception among citizens. Added to this picture is the fact that in recent years, there has been much more discourses introduced by the Brunei Government in relation to China, which includes the joint oil and gas explorations agreements and the convergence of grand strategies between the BRI and the *Wawasan Brunei 2035* ( Bimo, 2025; Bo, 2017; Teja, 2024; Yilmaz, 2025; Zhen, 2018; Zhou, 2025). Consequently, the lens that respondents see the issue is much more complex than that, for example, the lens of Vietnam and the Philippines that encounters the (near) daily presence of the Chinese law enforcement vessels within their maritime zones ( Chubb, 2022; Gurung, 2018; Heydarian, 2018; Pemmaraju, 2016; Sangtam, 2021). Nevertheless, more on Brunei’s perception of the South China Sea and China in general is presented in the following section as part of the top-down model interpretation.

### 4.2 The top-down model: How Brunei elites shape its citizens’ perceptions on the South China Sea issues

The central argument proposed in this section is that the public adopts the perspectives of the Brunei elites on foreign policy matters, which aligns with several past studies ( Boucher, 2024; Druckman, 2001; Western, 2005; Zaller, 1992). In the case of Brunei’s South China Sea policy, its claimant status has been marked by relative calm and a relaxed state, albeit with incursions by Chinese law enforcement vessels in the vast ( IMOA, 2020; Jacques, 2018; Putra, 2021, 2024; Sands, 2026). However, the view that the Sultanate is adopting is that the relations with China is too important to risk, and adopting a more decisive and confrontational response would undermine the Sultan’s attempts to solidify the trust of China as Brunei’s partner in achieving a more diversified economy in alignment to the *Wawasan Brunei 2035* ( Bo, 2017; CSPS, 2022; Koh, 2024; Tisdell, 1998; WB, 2008) *.*

How has this top-down model manifested in the form of Brunei’s public opinions towards the South China Sea? A look into the State of Southeast Asia report of 2025, with some comparisons with the numbers yielded in 2024, shows some unique features from the perspective of both Brunei in the context of the South China Sea, and Brunei’s relations with China (which ultimately affects the Brunei people’s perceptions of the disputed waters). Within the context of ‘Regional Outlook and Views on International Developments,’ the majority of Brunei respondents chose ‘China’s militarization and assertive actions in the South China Sea’ (51.0%) and ‘China’s encroachments in the exclusive economic zones and continental shelves of Southeast Asia’s littoral states’ ( Seah et al., 2025).

Perceptions of ASEAN’s role in the South China Sea are also unique for this section. Respondents had to choose two options on the question of how ASEAN should respond to the disputed waters; the majority of respondents chose the option that ASEAN must stand firm in holding its principles and align with relevant international laws, with a total of 63.1% secured voices from the Brunei people ( Seah et al., 2025). Similarly, there is a strong confidence expressed by the Brunei people over the potential effectiveness of the Code of Conduct, with 28.9% of respondents stating that its finalization would “[…] prevent other powers conducting military activities and energy exploration with ASEAN member states in the South China Sea” ( Seah et al., 2025, p. 26).

The respondents’ voices regarding the South China Sea and their perceptions of the nexus between ASEAN and the disputed waters are interesting to assess within the context of the top-down model. One conclusion is that the Brunei public expresses caution regarding China’s militarization and actions in the South China Sea. Does this mean that there is a misalignment with the Brunei elite’s perspective in handling the disputed waters? Not necessarily; in the past, Brunei has also expressed concerns about developments in the South China Sea ( Hart, 2018; IMOA, 2020; Putra, 2024). Therefore, although the Sultan would prefer a calm stance in the disputed waters, it does not neglect the fact that tensions in the South China Sea are concerning to the Brunei nation.

Furthermore, this favorable perception of ASEAN as a potential stakeholder in driving change in the South China Sea aligns with the top-down model in explaining the interaction between public opinion and foreign policy. The State of Southeast Asia report shows that Brunei citizens are happy with ASEAN being at the center of solutions related to the disputes. This perception, therefore, comes from above. In the past, Brunei elites have repeatedly stated that they stand by the relevance of ASEAN mechanisms in resolving tensions in the South China Sea ( ASEAN, 2012; Bukit & Sworn, 2021; Espena & Uy, 2020). For some scholars, for example, this has been described as a two-way solution, with one of the methods being the management of the South China Sea dispute through ASEAN’s regional approaches ( Espena & Uy, 2020).

Nevertheless, a look at Brunei’s public opinion will also reveal other important data related to the discussions in this section. Perceptions of the South China Sea should not be represented solely by questions that directly reference it. Alternatively, perceptions can be obtained from respondents who are asked about their perceptions of China in general. Therefore, the interpretation of the State of Southeast Asia survey report is divided into two categories. First, the answers that directly reference the South China Sea incidents (presented in the previous paragraphs). Second, the answers allow this article to frame the extent of trust the Brunei people have in China as an emerging power in the region. On the second category, there is a favorable perception embraced towards China, which goes align to the vast literature that has discussed Brunei’s close alignment with China in the economic realm ( CGTN, 2018; Druce & Julay, 2019; C.-Y. Hoon & Hashim, 2024; Huaxia, 2022; Kon, 2020; Lawrence, 2021; Zhao & Hoon, 2023).

The Sultan’s positive perspective towards China would eventually trickle down to the citizens’ embrace of it. For example, as seen in the Table 3 below, when asked who the most influential economic power in Southeast Asia is, the majority of respondents chose China with 51.7% in 2025 ( Seah et al., 2025). Interestingly, there is a significant gap in Brunei’s public opinion towards China and other potential economic powerhouses, such as ASEAN (16.1%) and the United States (11.4%) ( Seah et al., 2025). Interestingly, the follow-up question asked the respondent for their view on the economic powerhouse’s influence on Brunei. The survey showed that a steady number of voices were expressed, with 50.6% of Brunei respondents welcoming China’s regional economic influence ( Seah et al., 2025). Compared with the figures in 2024, this percentage declined only slightly from 51.0% ( Seah et al., 2024).

**Table 3.** Respondents’ views on the country/regional organization most influential in Southeast Asia based on economic power (2024 and 2025 comparison, in percentages).

ASEAN	2024	18.2%
	2025	16.1%
Australia	2024	1.3%
	2025	0.7%
China	2024	63.6%
	2025	51.7%
The European Union	2024	1.3%
	2025	2.0%
India	2024	1.3%
	2025	3.4%
Japan	2024	2.6%
	2025	9.4%
Republic of Korea	2024	0.0%
	2025	4.0%
The United States	2024	7.8%
	2025	11.4%
The United Kingdom	2024	3.9%
	2025	1.3%

Source: Adapted from the State of Southeast Asia 2025 and 2024 Survey Report ( Seah et al., 2024, 2025).

Meanwhile, among the Brunei public’s perceptions of the actor with the most strategic and political influence in Southeast Asia, China again ranked first. In 2025, respondents who chose China accounted for 31.5% of the Brunei respondents ( Seah et al., 2025). Interestingly, there still seems to be a balance of voices when respondents were asked about the actor’s political and strategic power’s influence in Brunei, with 51.1% expressing concern about its influence, while 48.9% welcoming it ( Seah et al., 2024, 2025).

The unique insights of Brunei’s public perceptions towards China do not stop there. When asked who ASEAN should align with if they had to choose between China and the US, surprisingly, the majority of Brunei respondents chose China. In the 2025 survey, 55.0% chose China, and 45.0% of respondents chose the United States ( Seah et al., 2025). Perhaps this is why there has been a favorable perception among the Brunei people. In one of the asked questions about how people perceived relations with China over the next three years (see Table 4), only a minority of respondents (1.3%) chose ‘worsen significantly,’ and 8.1% chose ‘worsen’ ( Seah et al., 2025). Meanwhile, the majority of respondents (38.3%) chose ‘improve,’ and 20.8% chose ‘improve significantly.’ This shows that the Brunei public’s perception of China is not isolated from its perception of the South China Sea, and people also view China from other perspectives, primarily from the perspective of China’s value as a firm partner for Brunei’s economic future.

**Table 4.** Respondents’ views on the direction of Brunei and China relations in the next three years (2024 and 2025 comparison, in percentages).

Worsen significantly		Worsen		Remain the same		Improve		Improve significantly	
2024	2025	2024	2025	2024	2025	2024	2025	2024	2025
5.2%	1.3%	10.4%	8.1%	31.2%	31.5%	40.3%	38.3%	13.0%	20.8%

Source: Adapted from the State of Southeast Asia 2025 and 2024 Survey Report ( Seah et al., 2024, 2025).

The last group of questions asks about trust towards China. In general, Brunei respondents are asked first whether they have confidence in China doing the right thing in the context of contributing to global peace and security. Interestingly, only a minority of respondents (4.7% and 14.8%) chose ‘no confidence’ and ‘little confidence’ ( Seah et al., 2025). Meanwhile, the majority opted for the options of ‘confident’ (34.9%) and ‘very confident’ (15.4%) ( Seah et al., 2025). Delving deep into the context of trust towards China, 29.3% of Brunei respondents selected the option of ‘China has vast economic resources and strong political will to provide global leadership’ when asked why they trusted China ( Seah et al., 2025, p. 58). Meanwhile, for those who expressed distrust, the main answer provided was concern that ‘China’s economic and military power could be used to threaten my country’s interests and sovereignty’ ( Seah et al., 2025).

The Brunei people’s public perceptions presented in this section are interesting to decipher. In alignment with the top-down model introduced by Boucher in 2024, the Bruneian people, as reflected in the State of Southeast Asia survey reports, show a clear adoption of the Sultan’s vision and perception of the South China Sea. There is a clear distinction in perceptions of the South China Sea and of China as a nation. In the context of the South China Sea, the public supports a stance that raises concerns about the aggression evolving in the disputed waters. However, there is still trust towards regional mechanisms as a means to manage the tensions. Similarly, although Brunei has overlapping maritime zones with China, this has not led to a negative perception of China in a broader sense. Looking at public trust in China as an economic powerhouse and an emerging nation, it is clear that public opinion favors China’s presence in the region, though it is challenged in the South China Sea dispute. These perceptions derive from the top and have been the Sultan’s South China Sea policies and perspectives for years.

## 5. Conclusion

As a claimant state in the South China Sea, Brunei Darussalam’s policy is puzzling. Despite having a rectangular EEZ, the Sultanate’s claimed maritime zones are not decisively safeguarded, and the disputed waters are not populated. For decades, scholars have interpreted this silence in Brunei’s South China Sea policy as reflecting economic considerations, leading Brunei to adopt a different stance than other claimant states. Nevertheless, is there a different angle of interpretation beyond those considerations?

By examining Brunei’s South China Sea policy, this study bridges the applicability and relevance of public opinion in foreign policy discourse. To understand what influences Brunei’s public opinion and the relationship between public opinion and foreign policy, this study argues for the relevance of Boucher’s 2024 conceptions. At the first level, Brunei’s public opinion on the disputed waters can be understood through the lens of factors influencing public foreign policy attitudes, which are shaped by socio-demographic factors and people’s conceptual schemes. Meanwhile, to understand the interaction between Brunei’s public opinion and the adopted South China Sea policy, this study argues for the relevance of the top-down model, in which Brunei’s elites shape the public’s understanding and preferences regarding the South China Sea. Brunei’s public opinion in this study draws on the published 2024 and 2025 surveys on the ‘State of Southeast Asia,’ which examine the views of 2,023 respondents on pressing regional matters affecting Southeast Asia.

By isolating Brunei respondents’ voices, several unique insights can be obtained. First, several variables are argued to affect the Brunei public’s perceptions of the South China Sea. The surveys indicated that the Brunei public is concerned about developments in the South China Sea, albeit not to the extent that it warrants emergency security responses. A look at the different socio-demographic factors at play shows that higher-educated respondents and the survey’s affiliations lead the public to adopt a more objective view of Brunei’s geopolitical challenges in Southeast Asia, which are not confined to those arising from the South China Sea alone. The balanced perspectives of the Brunei people can also be associated with Boucher’s conception of conceptual schemes/belief systems, as the public frames questions to fit their images and understanding of Southeast Asian regional dynamics. Therefore, the lens through which respondents see the South China Sea is much more complex than that through the security lens alone.

Furthermore, the interaction between public opinion and foreign policy can be seen in the alignment of the Sultanate’s and Brunei’s public opinion. At the first level, Brunei’s public opinion expresses concern over the development of the South China Sea and often emphasizes the importance of ASEAN-centered solutions to manage tensions. This aligns with the Sultan’s perspectives, in which, although it adopts a relatively calm stance in the South China Sea, it continues to emphasize the importance of solutions and the need for all conflicting actors to refrain from excessive actions. Furthermore, there is a clear leaning towards China in the context of public opinion on the economic powerhouse affecting Southeast Asia and the most influential actor in terms of strategic and political significance. This perception stems from the top, marked by the Sultan’s increasing alignment with China’s financial opportunities, which is seen as having the potential to diversify the state’s economy and align with Brunei’s grand strategy, *Wawasan Brunei 2035.*

## Ethical statement

This article does not require an ethical approval as it does not gather primary data from the respondents directly, and this case study is a qualitative analysis of published data by another source. Questions on ethical procedures and informed consent of participants from the utilized published data (State of Southeast Asia survey report) can be obtained from the ISEAS Yusof Ishak Institute.

## Data Availability

No underlying data are associated with this article. The data used for this study can be accessed online:
1. The State of Southeast Asia 2024 Survey Report ( Seah et al., 2024): https://www.iseas.edu.sg/centres/asean-studies-centre/state-of-southeast-asia-survey/the-state-of-southeast-asia-2024-survey-report/
2. The State of Southeast Asia 2025 Survey Report ( Seah et al., 2025): https://www.iseas.edu.sg/centres/asean-studies-centre/state-of-southeast-asia-survey/the-state-of-southeast-asia-2025-survey-report/
