# Supplementary material for: Revisiting the Role of Public Opinion in Foreign Policy: The Case of Brunei in the South China Sea
Source: F1000Res. 2026 May 16;15:417. Originally published 2026 Mar 19. [Version 2] doi: 10.12688/f1000research.177157.2 (PMC13231139; doi:10.12688/f1000research.177157.2)
Supplement: Peer review history

# Reviewer report: Reviewer response for version 2

**DOI:** 10.5256/f1000research.200621.r486127

**Published:** 2026-06-02

**Authors:** Pradeep Nair

**Affiliations:** 1 Sabin Center for Climate Change Law, Columbia University School of Law (Ringgold ID: 33804), New York, New York, USA; 2 Central University of Himachal Pradesh, Dharamshala, Himachal Pradesh, India

The study protocol is quite interesting and establishes the nexus between public opinion and foreign policy through the lens of South China Sea dispute but lacks clarity on how the various components of the dispute especially in terms of policy and governance approaches connects with the public opinion and foreign policies of Brunei. The study tried to look into how the gaps in existing knowledge about the process of influence factors of regional disputes has an overall impact on the diagnosis of the conflict of South China Sea, but miserably fails as it lacks a clear description of interpretation/analysis techniques – procedure and instruments; and also fails to justify the rationale of executing the study. The details of the usage of Diplomacy and Governance to understand the dispute lack procedural protocols. While the subject overall is worthwhile, the execution of the study makes it difficult to realize its importance.

 In this study there is a need to have a better weaving of the method and in-depth analysis of the public opinion acceptance and diffusion selected for study. The interpretation section is poor and lacks a clear description of the analytical framework used to conduct the study. The study design lacks essential details. The whole study is presented loosely and connecting points were missing. Further, what is missing is the theoretical contribution that the research forwards. Concluding remarks like an adherence is not sufficiently addressed in dispute resolution needs substantial evidential support.

 The conclusion is difficult to understand in terms of its connection with the interpretation and review of literature – this section needs work to make connections.

 Both the interpretation and conclusion sections need major revision. The literature and conclusions make some good points but need considerable editing.

Is the case presented with sufficient detail to be useful for teaching or other practitioners?

Partly

Is the work clearly and accurately presented and does it cite the current literature?

Partly

If applicable, is the statistical analysis and its interpretation appropriate?

Partly

Are all the source data underlying the results available to ensure full reproducibility?

Yes

Are the conclusions drawn adequately supported by the results?

Partly

Is the background of the case’s history and progression described in sufficient detail?

Partly

Reviewer Expertise:

climate change, capacity development, governance and science to policy

I confirm that I have read this submission and believe that I have an appropriate level of expertise to confirm that it is of an acceptable scientific standard, however I have significant reservations, as outlined above.

# Reviewer report: Reviewer response for version 2

**DOI:** 10.5256/f1000research.200621.r485500

**Published:** 2026-06-02

**Authors:** Mansur Juned

**Affiliations:** 1 Universitas Pembangunan Nasional Veteran Jakarta, Jakarta, Special Capital Region of Jakarta, Indonesia

Dear author. I have read your revised manuscript and have deliver the expected revision to make the article better and comprehensively i think it is ready to be indexed.

Is the case presented with sufficient detail to be useful for teaching or other practitioners?

Yes

Is the work clearly and accurately presented and does it cite the current literature?

Partly

If applicable, is the statistical analysis and its interpretation appropriate?

Partly

Are all the source data underlying the results available to ensure full reproducibility?

Yes

Are the conclusions drawn adequately supported by the results?

Partly

Is the background of the case’s history and progression described in sufficient detail?

Partly

Reviewer Expertise:

Geopolitics and Geoeconomics in Indo-Pacific

I confirm that I have read this submission and believe that I have an appropriate level of expertise to confirm that it is of an acceptable scientific standard.

# Reviewer report: Reviewer response for version 1

**DOI:** 10.5256/f1000research.195330.r471550

**Published:** 2026-04-20

**Authors:** Mai Quoc Dung

**Affiliations:** 1 Ho Chi Minh City University of Industry and Trade, Ho Chi Minh City, Ho Chi Minh City, Vietnam

**1. General Evaluation**

 The article provides a valuable and timely contribution to the study of Brunei’s foreign policy by shifting the focus from purely economic determinants to the role of public opinion. Exploring the domestic-foreign policy nexus in an absolute monarchy offers a refreshing perspective. The use of recent data from the “State of Southeast Asia” surveys (ISEAS-Yusof Ishak Institute) adds empirical weight to the discussion. However, several critical areas regarding methodology, theoretical depth, and comparative analysis require further refinement to meet the standards for full approval.

**2. Specific Comments and Requirements for Revision**

 Methodology and Data Representation

 The ISEAS survey data primarily reflects the views of regional experts, officials, and stakeholders (the “elite”). The author must explicitly discuss whether these findings can be generalized as “public opinion” in the broader sense within Brunei. If the data is restricted to a certain social stratum, the author should adjust the terminology to “elite opinion” or provide a justification for why this group’s views serve as a reliable proxy for the general populace.

 While Figures 1 through 4 provide essential statistics, the analysis remains somewhat descriptive. The author is encouraged to interpret these figures more deeply by linking specific percentage shifts to contemporary geopolitical events occurring between 2024 and 2025.

 Theoretical Framework and Mechanism

 The paper argues for a convergence between the state and the people. However, the “black box” of how this consensus is formed remains unopened. The author should elaborate on the mechanisms (e.g., state-controlled media, educational curricula, or the “Malay Islamic Monarchy” ideology) that align public perception with the Sultan’s strategic vision.

 Does the public support the government's “quiet diplomacy” because they actively agree with the strategy, or because the South China Sea issue is perceived as a low-priority concern compared to domestic welfare? Clarifying the salience of this issue in the public mind would strengthen the argument.

 Comparative and Contextual Analysis

 To highlight the uniqueness of the Bruneian case, a brief comparative reference to other ASEAN claimants (e.g., the Philippines or Vietnam) would be beneficial. Why does similar Chinese assertiveness lead to public outcry in Manila but “strategic trust” in Bandar Seri Begawan?

 The author seeks to provide an alternative to the “economic dependency” thesis. To do this effectively, the revision should more clearly demonstrate where public opinion complements or diverges from purely economic motives.

**3. Conclusion**

 The manuscript is well-written and addresses a significant gap in the literature. However, the transition from descriptive data to analytical conclusions needs to be more robust. By addressing the representation of the survey sample and the mechanisms of top-down consensus, the author will significantly enhance the scholarly impact of this work.

Is the case presented with sufficient detail to be useful for teaching or other practitioners?

Partly

Is the work clearly and accurately presented and does it cite the current literature?

Partly

If applicable, is the statistical analysis and its interpretation appropriate?

Partly

Are all the source data underlying the results available to ensure full reproducibility?

Partly

Are the conclusions drawn adequately supported by the results?

Partly

Is the background of the case’s history and progression described in sufficient detail?

Partly

Reviewer Expertise:

History, Political Science, International Relations and Regional Relations

I confirm that I have read this submission and believe that I have an appropriate level of expertise to confirm that it is of an acceptable scientific standard, however I have significant reservations, as outlined above.

# Response

**Published:** 2026-04-29

**Authors:** bama andika putra

**Affiliations:** University of Bristol School of Sociology Politics and International Studies, Bristol, England, UK

**Comment 1:** The ISEAS survey data primarily reflects the views of regional experts, officials, and stakeholders (the “elite”). The author must explicitly discuss whether these findings can be generalized as “public opinion” in the broader sense within Brunei. If the data is restricted to a certain social stratum, the author should adjust the terminology to “elite opinion” or provide a justification for why this group’s views serve as a reliable proxy for the general populace.

 Response: I agree with this suggestion that the elites and public opinion variables require further elaboration. Two responses:

 1. On the question of elites: There are different opinions as to what constitutes elites within the country. As my study focuses on the nexus between public opinion and foreign policy, elites are defined based on Boucher’s 2024 study, which includes those in higher hierarchical positions (government officials). 

 2. On public opinion: The structure of public opinion, as added in the new paragraph in section 3, looks at how the social categories used in the ISEAS survey (academics, business people, etc.) also fall under the category of public opinion. First, I cited several studies that looked at the different backgrounds that past studies have understood as constituting the public. Second, I explain how these respondents are best described as ‘informed public,’ meaning they have a better understanding of what is happening within the context of Brunei’s foreign policies. I went on to cite several studies that also argue for the benefits of using informed public voices rather than uninformed ones, given the risk of being easily misled by misinformation and the instability of policy preferences. The added paragraph (page 7): “In the context of the surveys utilized for this study, including academics, business actors, government, and non-government workers, do not fall under the category of elites within the definition of Boucher’s 2024 study, but are part of a group of informed individuals on international relations issues. Therefore, past discourses have also classified these backgrounds as part of public opinion (Harrison, 2022; Nyberg & Murray, 2023; Püttmann et al., 2022), providing a unique angle of perspective of matters being questioned. Being informed is part of the public, and this provides an advantage for the interpretations offered in this study, as uninformed opinions have been framed in past studies as unable to hold firm policy preferences and as easily being misled by misinformation (Mayeur et al., 2024; Panish, 2025; Schäfer & Schemer, 2024).”

**Comment 2** : While Figures 1 through 4 provide essential statistics, the analysis remains somewhat descriptive. The author is encouraged to interpret these figures more deeply by linking specific percentage shifts to contemporary geopolitical events occurring between 2024 and 2025.

 Response: The structure of the discussions follows a particular sequence: Explanation of the geopolitical events that occurred in 2024 and 2025, which are then followed by Brunei’s respondents’ perspectives on actors or issues related to that. This is clearly displayed in the third paragraph revision (listed below) that explains that Brunei continue to face intrusions in the maritime sector, increased pressure to realize the Wawasan Brunei 2035 by diversifying its economy, and the dilemma of being too aligned to Brunei. Meanwhile, the first two listed revised paragraphs below fulfill the Reviewer 2’s recommendation, by directly mentioning the empirical case related to the results of the survey at that time. The revisions: 

 1: Paragraph 2, page 9: “In a more specific question, respondents are pushed to select three geopolitical events that are the primary concern of their country. Brunei’s public opinion in the 2025 report showed that two geopolitical events have been the main concerns for the Brunei Government: the war between Israel and Palestine (56.4%) and the aggressive behavior in the South China Sea (57.7%) ( Seah et al., 2025). A unique observation is that respondents who selected the South China Sea issue were mainly from the ASEAN claimant states, including the Philippines (90.3%), Vietnam (74.8%), and Brunei (57.7%) ( Seah et al., 2025, p. 16). This came in a time where an increased assertiveness is observed in the disputed waters of the South China Sea, marked with the vast incursions made into the the claimant state’s waters (Joshi & Tara Singh, 2021; Raymond & Welch, 2022). Nevertheless, these figures from 2025 show that Brunei citizens’ perception of the significance of aggressive behavior in the South China Sea is significantly lower than that of other claimant states.” 

 2. Paragraph 2, page 10: “Several survey results from 2024 and 2025 reveal unique insights that allow this study to examine the relevance of several variables in shaping the Brunei public’s perceptions. The first noticeable thing is that respondents do not downplay issues related to China and the South China Sea, indicating they acknowledge that tensions exist and pose a challenge for the Brunei Government. Those general perceptions themselves mimic those of the Sultanate, in which Brunei confronts multiple security challenges in the region, and the South China Sea is only one among many ( Kurniati et al., 2025; Lee et al., 2024; Shaw, 2016; Widyawardhana et al., 2018). Furthermore, the respondents also express that the South China Sea issue is present but not a significant concern, unlike the voices expressed in the cases of Vietnam and the Philippines. This is also a similar stance to the Sultan, which perceives that the South China Sea is a challenge, but does not require excessive responses in the form of populating the disputed waters ( IMOA, 2020; Kurniati et al., 2025; Noor & Daniel, 2016; Putra, 2021; Roach, 2014; Xinhua, 2016; Zhou, 2025), despite the maritime tensions continues to take place in recent years. ”

 3. Page 11, paragraph 5: “The central argument in this section is that there is a correlation between the perspectives of the Brunei elite on foreign policy matters and those of the public, which aligns with several past studies (Boucher, 2024; Druckman, 2001; Western, 2005; Zaller, 1992). In the case of Brunei’s South China Sea policy, its claimant status has been marked by relative calm and a relaxed state, albeit with incursions by Chinese law enforcement vessels in the vast ( IMOA, 2020; Jacques, 2018; Putra, 2021, 2024; Sands, 2026). However, the view that the Sultanate is adopting is that the relations with China is too important to risk, and adopting a more decisive and confrontational response would undermine the Sultan’s attempts to solidify the trust of China as Brunei’s partner in achieving a more diversified economy in alignment to the Wawasan Brunei 2035 ( Bo, 2017; CSPS, 2022; Koh, 2024; Tisdell, 1998; WB, 2008) . ”

**Comment 3** : The paper argues for a convergence between the state and the people. However, the “black box” of how this consensus is formed remains unopened. The author should elaborate on the mechanisms (e.g., state-controlled media, educational curricula, or the “Malay Islamic Monarchy” ideology) that align public perception with the Sultan’s strategic vision.

 Does the public support the government's “quiet diplomacy” because they actively agree with the strategy, or because the South China Sea issue is perceived as a low-priority concern compared to domestic welfare? Clarifying the salience of this issue in the public mind would strengthen the argument.

 Response: I explore several variables that would facilitate convergence in perceptions between the state and the people. First, the revision examines state-controlled media in Brunei, noting a hierarchical structure in the information disseminated to the public due to family ownership. Second, it points to the unique educational system in Brunei, which ensures that the Sultan’s form of governance (including the Malay Islamic Monarchy’s state philosophy) is respected and internalized by the public. The added paragraph (page 11, paragraph 2): “Regarding the variables that influence this, several variables can be identified. A look at public-state discourse in Brunei reveals that the Sultanate has historically communicated its foreign policy priorities to its populace. For one, state-controlled media in Brunei is strong. State-owned Radio Television Brunei and the daily newspaper (the Borneo Bulletin) are controlled by the Sultan’s family, allowing the Sultan’s preferences to be voiced to the public (FH, 2024; RSF, 2026). Brunei officials can also close news media outlets for reporting deemed by the Sultanate to be misleading or false, thereby allowing full control over public discourse. Furthermore, education in Brunei has ensured that it aligns with the Sultan’s way of governance, reforming the education system to ensure a blend of Bruneian identity and culture, and the Malay Islamic Monarchy being upheld as the state’s philosophy (Mohamad et al., 2018; Muhammad & Petra, 2021; Müller, 2025)”

**Comment 4** :  To highlight the uniqueness of the Bruneian case, a brief comparative reference to other ASEAN claimants (e.g., the Philippines or Vietnam) would be beneficial. Why does similar Chinese assertiveness lead to public outcry in Manila but “strategic trust” in Bandar Seri Begawan?

 The author seeks to provide an alternative to the “economic dependency” thesis. To do this effectively, the revision should more clearly demonstrate where public opinion complements or diverges from purely economic motives.

 Revision 1: I revised the paragraph on the conceptual schemes/belief systems (page 11, last paragraph) to explain how the Vietnam and the Philippine cases differ from Brunei due to the extent of China’s maritime presence within the disputed EEZ. Due to the vastness of the maritime claims held by those two nations that conflict with China’s Nine-Dash Line, they have continued to challenge China by exploring oil and gas reserves and increasing their maritime presence. This, however, is met with continued retaliation by China’s maritime constabulary forces. The revised paragraph: “Given the variable of conceptual schemes/belief systems, as argued previously, there is a likelihood that respondents frame the questions to fit their images and understandings ( Boucher, 2024; Holsti, 1962). The relatively balanced perception of Brunei citizens can be attributed to the fact that, although incursions by Chinese law enforcement vessels occurred in the past ( AMTI, 2020), this is not a continuing development that warrants a sustained threat perception among citizens. Added to this picture is the fact that in recent years, there has been much more discourses introduced by the Brunei Government in relation to China, which includes the joint oil and gas explorations agreements and the convergence of grand strategies between the BRI and the Wawasan Brunei 2035 ( Bimo, 2025; Bo, 2017; Teja, 2024; Yilmaz, 2025; Zhen, 2018; Zhou, 2025). Consequently, the lens that respondents see the issue is potentially much more complex than that, for example, the lens of Vietnam and the Philippines that encounters the (near) daily presence of the Chinese law enforcement vessels within their maritime zones ( Chubb, 2022; Gurung, 2018; Heydarian, 2018; Pemmaraju, 2016; Sangtam, 2021). These states have high stakes in the South China Sea, and their continued presence within their maritime borders and exploration of potential oil and gas reserves have been met with China’s increased assertiveness through the deployment of non-military vessels in overlapping EEZs with Vietnam and the Philippines. Nevertheless, more on Brunei’s perception of the South China Sea and China in general is presented in the following section as part of the top-down model interpretation.”

 Response 2: Besides economic factors, the revisions also focus on the perceptions of China from a political standpoint. In one of the respondent survey questions, the Brunei public expressed their perceptions that China is a country holding great political influence over the Southeast Asian region (going beyond economic discourses). The revised paragraph (Paragraph 1, page 14): “Meanwhile, among the Brunei public’s perceptions of the actor with the most strategic and political influence in Southeast Asia, China again ranked first. In 2025, respondents who chose China accounted for 31.5% of the Brunei respondents ( Seah et al., 2025). Interestingly, there still seems to be a balance of voices when respondents were asked about the actor’s political and strategic power’s influence in Brunei, with 51.1% expressing concern about its influence, while 48.9% welcoming it ( Seah et al., 2024, 2025). The respondent’s voices signal that the perception towards China goes beyond China’s perceived capacity in economic influence, as it is also perceived as influencing the politics in Southeast Asia. “

**Comment 5** : The manuscript is well-written and addresses a significant gap in the literature. However, the transition from descriptive data to analytical conclusions needs to be more robust. By addressing the survey sample's representation and the mechanisms of top-down consensus, the author will significantly enhance this work's scholarly impact.

 Response 1: The representation of the survey sample is provided in the revisions of section 3 (page 7).

 Response 2: The statistical interpretation in the revised version of the article represents correlation, not causation. As also suggested by Reviewer 1, I have pointed this out in several parts of the article and exemplify the point in the revised conclusions (see below). Therefore, I argue that the survey data captures a snapshot of sentiment, not the actual tracing of the transmission mechanisms through which the Sultanate shapes public voices (although this point is slightly elaborated in the argument on media control in Brunei). I also emphasize in the conclusion that the data complements the top-down model rather than definitively proving it. Paragraphs 2 and 3 of the revised conclusions (pages 15 and 16):

 Paragraph 2: “By examining Brunei’s South China Sea policy, this study argues that public opinion correlates with foreign policy. To understand what influences Brunei’s public opinion and the relationship between public opinion and foreign policy, this study argues for the relevance of Boucher’s 2024 conceptions. At the first level, Brunei’s public opinion on the disputed waters can be understood through the lens of factors influencing public foreign policy attitudes, which are shaped by socio-demographic factors and people’s conceptual schemes. Meanwhile, to understand the interaction between Brunei’s public opinion and the adopted South China Sea policy, this study argues for the relevance of the top-down model, in which Brunei’s elites shape the public’s understanding and preferences regarding the South China Sea. Brunei’s public opinion in this study draws on the published 2024 and 2025 surveys on the ‘State of Southeast Asia,’ which examine the views of 2,023 respondents on pressing regional matters affecting Southeast Asia.”

 Paragraph 3: “By isolating the voices of Brunei respondents, several unique insights can be obtained, which complement the top-down model assessed in this study. First, several variables are argued to affect the Brunei public’s perceptions of the South China Sea. The surveys indicated that the Brunei public is concerned about developments in the South China Sea, albeit not to the extent that it warrants emergency security responses. A look at the different socio-demographic factors at play shows that higher-educated respondents and the survey’s affiliations lead the public to adopt a more objective view of Brunei’s geopolitical challenges in Southeast Asia, which are not confined to those arising from the South China Sea alone. The balanced perspectives of the Brunei people can also be associated with Boucher’s conception of conceptual schemes/belief systems, as the public frames questions to fit their images and understanding of Southeast Asian regional dynamics. Therefore, the lens through which respondents see the South China Sea is much more complex than that through the security lens alone.”

# Reviewer report: Reviewer response for version 1

**DOI:** 10.5256/f1000research.195330.r471549

**Published:** 2026-04-14

**Authors:** Mansur Juned

**Affiliations:** 1 Universitas Pembangunan Nasional Veteran Jakarta, Jakarta, Special Capital Region of Jakarta, Indonesia

The manuscript presents a theoretically ambitious attempt to introduce domestic-level variables, specifically public opinion, into the analysis of Brunei’s foreign policy regarding the South China Sea (SCS) and China perspective in general, particularly the prospect of the future economic relations between two countries. By utilizing Jean-Christophe Boucher’s framework, the author attempts to move beyond the standard material and economic explanations for Brunei’s "silent claim."

 However, I perceived that the manuscript struggles to justify the causal weight assigned to public opinion in an absolute monarchy confronting external systemic pressures. While the author successfully demonstrates a correlation between state policy and public sentiment, it fails to provide a convincing explanation that public opinion acts as direct causal relations with elite decision-making. Instead, the data more accurately describes an engineered domestic consensus that reflects the state's material imperatives (depleting hydrocarbons and reliance on China's economic apparatus). The manuscript is engaging and well-written, but its causal claims require significant tempering.

**1. Is the background of the case’s history and progression described in sufficient detail?**

 The geopolitical and geoeconomic context is established with commendable precision. The author accurately maps Brunei’s material vulnerabilities, specifically its depleting hydrocarbon reserves and the subsequent pivot toward Wawasan Brunei 2035. The contrast between Brunei’s non-militarized maritime posture and the assertive tactics of neighboring littoral states is sharply defined.

 However, the manuscript requires a deeper historical baseline regarding the operational realities of public-state discourse in Brunei. To effectively argue a "top-down" model, the author must establish how the Sultanate has historically signaled foreign policy priorities to its populace. Without this institutional context, the sudden introduction of public survey data lacks a foundational anchor.

**2. Is the work clearly and accurately presented and does it cite the current literature?**

 The literature review exhibits a coherent synthesis, effectively triangulating three critical discourses: the material substance of territorial claims, the strategic calculus underpinning the "silent claim," and the comprehensive geoeconomic alignment with China. The integration of contemporary scholarship pertaining to dual alignment and hedging strategies within the Southeast Asian context is executed with commendable accuracy.

 However, please rearrange table 3 regarding “view of this political and strategic power’s influence on your country?” to display full context as it presented in the source to prevent misleading regarding the number. On the source it is stated the number of those who perceive positively and negatively. As well as other tables from the SEAH as it would be difficult for the reader to comprehend if the table did not display full information as it presented on the sources. Second, please provide a more robust explanation regarding the correlations of how the correlational relations between the public opinion and foreign policy of Brunei worked by providing a brief example to other issues to justify the pattern of Top-Down the manuscript mentions. 

**3. If applicable, is the statistical analysis and its interpretation appropriate?**

 The author relies entirely on descriptive statistics (frequency percentages) extracted from the 2024 and 2025 State of Southeast Asia reports.

 Critique for Refinement: Inferring complex conceptual schemes and causal interactions from basic frequency distributions is an analytical overreach.the statistical interpretation must be revised to reflect correlation, not causation, since it rely on the secondary statistical data that limit the room for further deepening or statistical method improvement since the raw data is not available. Conversely, the manuscript would be beneficial if the existing literature supports the correlation relations between Brunei’s public perception on China and the foreign policy particularly regarding how this result could occur.

**4. Are all the source data underlying the results available to ensure full reproducibility?**

 Yes. The data sourcing is highly transparent. The reliance on the ISEAS-Yusof Ishak Institute datasets is clearly documented, and the provision of direct URLs ensures that peer researchers can verify the sample size and reproduce the descriptive tables

**5. Are the conclusions drawn adequately supported by the results?**

 The descriptive conclusions regarding the current state of Bruneian public opinion are supported by the data: the populace views the SCS disputes with caution but retains high confidence in ASEAN's regional mechanisms and China's economic utility.

 Critique for Refinement: The primary conclusion—that elite cueing definitively shapes this public preference—is logically sound but empirically unsupported by the provided dataset. The survey data captures a snapshot of sentiment; it does not trace the transmission mechanism from the Sultanate to the public. In a state characterized by massive structural asymmetry with China, this public alignment is likely a pragmatic reflection of the state's survival strategy rather than a dynamic that "influences" foreign policy. The author must explicitly state that the data complements the top-down model rather than definitively proving it.

**6. Is the case presented with sufficient detail to be useful for teaching or other practitioners?**

 The manuscript exhibits substantial utility for graduate-level pedagogical frameworks and practitioners engaged in maritime security governance. It functions as a commendable and provocative case study for interrogating the applicability of domestic-level theoretical variables within non-Western, non-democratic institutional contexts. Furthermore, the systematic mapping of overlapping Exclusive Economic Zones (EEZs) provides commendable clarity for practitioners analyzing the geostrategic complexities within the ASEAN theater.

Is the case presented with sufficient detail to be useful for teaching or other practitioners?

Yes

Is the work clearly and accurately presented and does it cite the current literature?

Partly

If applicable, is the statistical analysis and its interpretation appropriate?

Partly

Are all the source data underlying the results available to ensure full reproducibility?

Yes

Are the conclusions drawn adequately supported by the results?

Partly

Is the background of the case’s history and progression described in sufficient detail?

Partly

Reviewer Expertise:

Geopolitics and Geoeconomics in Indo-Pacific

I confirm that I have read this submission and believe that I have an appropriate level of expertise to confirm that it is of an acceptable scientific standard, however I have significant reservations, as outlined above.

# Response

**Published:** 2026-04-29

**Authors:** bama andika putra

**Affiliations:** University of Bristol School of Sociology Politics and International Studies, Bristol, England, UK

**Comment 1** : The manuscript requires a deeper historical baseline regarding the operational realities of public-state discourse in Brunei. To effectively argue a "top-down" model, the author must establish how the Sultanate has historically signaled foreign policy priorities to its populace. Without this institutional context, the sudden introduction of public survey data lacks a foundational anchor.

 Response: I agree with this suggestion. To provide a baseline for the realities of Brunei’s public-state discourse, I have added a paragraph on page 11 to explain that it is structured hierarchically. This is marked by state-controlled media in Brunei and laws that prevent the spread of news deemed misleading. Therefore, the Sultanate has been using the platform to disseminate information on the Sultan’s South China Sea policies, including justifications for the stance taken. I added this paragraph before the explanation of the socio-demographic factors that influence the Brunei respondents’ perceptions vis-à-vis the South China Sea. The added paragraph: “Regarding the variables that influence this, several conclusions can be made. A look at public-state discourse in Brunei reveals that the Sultanate has historically communicated its foreign policy priorities to its populace. For one, state-controlled media in Brunei is strong. State-owned Radio Television Brunei and the daily newspaper (the Borneo Bulletin) are controlled by the Sultan’s family, allowing the Sultan’s preferences to be voiced to the public (FH, 2024; RSF, 2026). Brunei officials can also close news media outlets for reporting news deemed by the Sultanate to be misleading or false, allowing for full control over public discourse.”

**Comment 2** : However, please rearrange table 3 regarding “view of this political and strategic power’s influence on your country?” to display full context as it presented in the source to prevent misleading regarding the number. On the source it is stated the number of those who perceive positively and negatively. As well as other tables from the SEAH as it would be difficult for the reader to comprehend if the table did not display full information as it presented on the sources. 

 Response: For clarification, Table 3 is available on page 33 of the 2025 State of Southeast Asia report. The report asked: “In your view, which country/regional organization is the most influential economic power in Southeast Asia?” Respondents are given the option of several actors (countries and regional organizations) to choose from, and are not questioned whether they perceived the actors in a negative or positive light. This explains why there are countries like the Republic of Korea that attained a 0% response rate, meaning no respondents chose it as having significant economic influence in Southeast Asia. Nevertheless, I have made two changes, both the explanation of table 3, and the title of the table (pages 12-13): “The Sultan’s positive perspective towards China would eventually trickle down to the citizens’ embrace of it. For example, as shown in Table 3 below, when asked who the most influential economic power in Southeast Asia is, the majority of respondents chose China, with 51.7% in 2025 (Seah et al., 2025). Interestingly, there is a significant gap in Brunei’s public opinion towards China and other potential economic powerhouses, such as ASEAN (16.1%) and the United States (11.4%) ( Seah et al., 2025). The follow-up question asked the respondents for their view on the economic powerhouse’s influence on Brunei. The survey showed that a steady number of voices were expressed, with 50.6% of Brunei respondents welcoming China’s regional economic influence ( Seah et al., 2025). Compared with the figures in 2024, this percentage declined only slightly from 51.0% ( Seah et al., 2024).”

 I have also looked at the other tables and see no issues with the data details. Table 2 displays the respondents' preferences for who they perceived as an actor able to provide leadership in maintaining a rules-based order (page 41 of the 2025 State of Southeast Asia report). Table 4’s data is on page 48 of the ISEAS report, and the same data is presented in the original source. The only difference (for Tables 2, 3, and 4) is that it does not display the results of respondents from other countries in Southeast Asia. This is done on purpose, as including those figures would create confusion for readers, as they would not know where to place their focus. 

**Comment 3** : Second, please provide a more robust explanation regarding the correlations of how the correlational relations between the public opinion and foreign policy of Brunei worked by providing a brief example to other issues to justify the pattern of Top-Down the manuscript mentions. 

 Response: Other issues similar to the top-down interpretation of the public's and the Sultan’s perceptions of the South China Sea are evident in the public's positive sentiments towards ASEAN. As seen in the revisions to paragraph 3 (page 12), the Sultanate has in the past placed its faith in ASEAN mechanisms to maintain order in the South China Sea dispute. Having examined the public's favorable responses, this shows alignment of opinions, making the top-down interpretation viable. The revised paragraph: “Furthermore, this favorable perception of ASEAN as a potential stakeholder in driving change in the South China Sea aligns with the top-down model in explaining the interaction between public opinion and foreign policy. The State of Southeast Asia report shows that Brunei citizens are happy with ASEAN being at the center of solutions related to the disputes. This perception, therefore, comes from above. In the past, Brunei elites have repeatedly stated that they stand by the relevance of ASEAN mechanisms in resolving tensions in the South China Sea ( ASEAN, 2012; Bukit & Sworn, 2021; Espena & Uy, 2020). For some scholars, for example, this has been described as a two-way solution, with one of the methods being the management of the South China Sea dispute through ASEAN’s regional approaches ( Espena & Uy, 2020). Being less informed about the dynamics of ASEAN-Brunei relations, the top-down approach helps explain the emergence of the positive sentiments towards Brunei as being influenced by the Sultanate’s continuous engagements with the regional organization.”

**Comment 4** : The statistical interpretation must be revised to reflect correlation, not causation, since it rely on the secondary statistical data that limit the room for further deepening or statistical method improvement since the raw data is not available. Conversely, the manuscript would be beneficial if the existing literature supports the correlation relations between Brunei’s public perception on China and the foreign policy particularly regarding how this result could occur.

 Response: I agree with this recommendation. To avoid causation and focus the rhetoric on correlation, I have revised several parts of the discussion section as listed in the following:

 1. Page 7, last paragraph: “To reveal public opinion among Brunei citizens regarding the South China Sea, this section will be structured as follows. First, it provides information on the respondents in the 2025 survey report, including variables such as age and education, to provide background on them. Second, it elaborates on several key findings and opinions under the themes of regional outlook on international developments, major power influences, US-China rivalry in Southeast Asia, and perceptions of trust, which are correlated with the dynamics in the South China Sea. Third, through Boucher’s analytical frameworks that reveal factors influencing foreign policy attitude (age, education, and conceptual schemes/belief systems) and the form of interaction between the public’s foreign policy opinions and decision makers, this study explores how these two frameworks are applicable in understanding the position and role of public views in the context of Brunei’s South China Sea policy. Within this framework, this study measures and delineates the correlational relationship between public opinion and the eventual foreign policy direction taken by Brunei.”

 2. page 11, paragraph 1: “Furthermore, as argued with Boucher’s analytical framework, the socio-demographic factors are influential in the respondent’s perceptions as they are variables that are “[…] proxies for a deep-seated socialization process and gained experience which shapes attitudes on policy issues, and results are relatively consistent across foreign policy issues” ( Boucher, 2024, p. 252). Seen, for example, how Brunei citizens express a relatively equal concern for different geopolitical challenges in Asia, which are not confined to the South China Sea, the socio-demographic variables of education and affiliation background relates to the balanced perception held among Brunei citizens.” 

 3. page 11, paragraph 2: “Given the variable of conceptual schemes/belief systems, as argued previously, there is a likelihood that respondents frame the questions to fit their images and understandings ( Boucher, 2024; Holsti, 1962). The relatively balanced perception of Brunei citizens can be attributed to the fact that, although incursions by Chinese law enforcement vessels occurred in the past ( AMTI, 2020), this is not a continuing development that warrants a sustained threat perception among citizens. Added to this picture is the fact that in recent years, there has been much more discourses introduced by the Brunei Government in relation to China, which includes the joint oil and gas explorations agreements and the convergence of grand strategies between the BRI and the Wawasan Brunei 2035 ( Bimo, 2025; Bo, 2017; Teja, 2024; Yilmaz, 2025; Zhen, 2018; Zhou, 2025). Consequently, the lens that respondents see the issue is potentially much more complex than that, for example, the lens of Vietnam and the Philippines that encounters the (near) daily presence of the Chinese law enforcement vessels within their maritime zones ( Chubb, 2022; Gurung, 2018; Heydarian, 2018; Pemmaraju, 2016; Sangtam, 2021). Nevertheless, more on Brunei’s perception of the South China Sea and China in general is presented in the following section as part of the top-down model interpretation.”

 4. Page 11, paragraph 3: “The central argument in this section is that there is a correlation between the perspectives of the Brunei elite on foreign policy matters and those of the public, which aligns with several past studies (Boucher, 2024; Druckman, 2001; Western, 2005; Zaller, 1992). In the case of Brunei’s South China Sea policy, its claimant status has been marked by relative calm and a relaxed posture, albeit with incursions by Chinese law enforcement vessels in the vast (; Jacques, 2018; Putra, 2021, 2024; Sands, 2026). However, the view that the Sultanate is adopting is that the relations with China is too important to risk, and adopting a more decisive and confrontational response would undermine the Sultan’s attempts to solidify the trust of China as Brunei’s partner in achieving a more diversified economy in alignment to the Wawasan Brunei 2035 ( Bo, 2017; CSPS, 2022; Koh, 2024; Tisdell, 1998; WB, 2008) .”

 5. page 14, paragraph 3: “The Brunei people’s public perceptions presented in this section are interesting to decipher. In line with the top-down model introduced by Boucher in 2024, the Bruneian people, as reflected in the State of Southeast Asia survey reports, show a correlation to the Sultan’s vision and perception of the South China Sea. There is a clear distinction in perceptions of the South China Sea and of China as a nation. In the context of the South China Sea, the public supports a stance that raises concerns about the aggression evolving in the disputed waters. However, there is still trust towards regional mechanisms as a means to manage the tensions. Similarly, although Brunei has overlapping maritime zones with China, this has not led to a negative perception of China in a broader sense. Given public trust in China as an economic powerhouse and emerging nation, it is clear that public opinion favors China’s presence in the region, though it is challenged in the South China Sea dispute.”

 6. page 15, paragraph 2: “By examining Brunei’s South China Sea policy, this study argues that public opinion correlates with foreign policy. To understand what influences Brunei’s public opinion and the relationship between public opinion and foreign policy, this study argues for the relevance of Boucher’s 2024 conceptions. At the first level, Brunei’s public opinion on the disputed waters can be understood through the lens of factors influencing public foreign policy attitudes, which are shaped by socio-demographic factors and people’s conceptual schemes. Meanwhile, to understand the interaction between Brunei’s public opinion and the adopted South China Sea policy, this study argues for the relevance of the top-down model, in which Brunei’s elites shape the public’s understanding and preferences regarding the South China Sea. Brunei’s public opinion in this study draws on the published 2024 and 2025 surveys on the ‘State of Southeast Asia,’ which examine the views of 2,023 respondents on pressing regional matters affecting Southeast Asia.”

**Comment 5** : Are the conclusions drawn adequately supported by the results? The primary conclusion—that elite cueing definitively shapes this public preference—is logically sound but empirically unsupported by the provided dataset. The survey data captures a snapshot of sentiment; it does not trace the transmission mechanism from the Sultanate to the public. In a state characterized by massive structural asymmetry with China, this public alignment is likely a pragmatic reflection of the state's survival strategy rather than a dynamic that "influences" foreign policy. The author must explicitly state that the data complements the top-down model rather than definitively proving it.

 Response: I agree with the recommendations. In the last three paragraphs of the article (the conclusion section), I revised the words to be more cautious. Rather than arguing for causality, I argue that the analysis is evident from a correlation between public opinion discourse and foreign policy decisions. The revisions: 

 1. Paragraph 2, page 15: “By examining Brunei’s South China Sea policy, this study argues that public opinion correlates with foreign policy. To understand what influences Brunei’s public opinion and the relationship between public opinion and foreign policy, this study argues for the relevance of Boucher’s 2024 conceptions. At the first level, Brunei’s public opinion on the disputed waters can be understood through the lens of factors influencing public foreign policy attitudes, which are shaped by socio-demographic factors and people’s conceptual schemes. Meanwhile, to understand the interaction between Brunei’s public opinion and the adopted South China Sea policy, this study argues for the relevance of the top-down model, in which Brunei’s elites shape the public’s understanding and preferences regarding the South China Sea. Brunei’s public opinion in this study draws on the published 2024 and 2025 surveys on the ‘State of Southeast Asia,’ which examine the views of 2,023 respondents on pressing regional matters affecting Southeast Asia. 

 2. Paragraph 3, page 15: By isolating the voices of Brunei respondents, several unique insights can be obtained, which complement the top-down model assessed in this study. First, several variables are argued to affect the Brunei public’s perceptions of the South China Sea. The surveys indicated that the Brunei public is concerned about developments in the South China Sea, albeit not to the extent that it warrants emergency security responses. A look at the different socio-demographic factors at play shows that higher-educated respondents and the survey’s affiliations lead the public to adopt a more objective view of Brunei’s geopolitical challenges in Southeast Asia, which are not confined to those arising from the South China Sea alone. The balanced perspectives of the Brunei people can also be associated with Boucher’s conception of conceptual schemes/belief systems, as the public frames questions to fit their images and understanding of Southeast Asian regional dynamics. Therefore, the lens through which respondents see the South China Sea is much more complex than that through the security lens alone.

 3. Paragraph 4, page 15: Furthermore, the interaction between public opinion and foreign policy is evident in the alignment of the Sultanate’s and Brunei’s public opinion. At the first level, Brunei’s public opinion expresses concern about developments in the South China Sea and often emphasizes the importance of ASEAN-centered solutions to manage tensions. This correlates with the Sultan’s perspectives, in which, although it adopts a relatively calm stance in the South China Sea, it continues to emphasize the importance of solutions and the need for all conflicting actors to refrain from excessive actions. Furthermore, there is a clear leaning towards China in public opinion on the economic powerhouse affecting Southeast Asia, and the most influential actor in terms of strategic and political significance. This perception potentially stems from the top, marked by the Sultan’s increasing alignment with China’s financial opportunities, which is seen as having the potential to diversify the state’s economy and align with Brunei’s grand strategy, Wawasan Brunei 2035.”
